# JNK2 Promotes Endothelial Cell Alignment under Flow

**DOI:** 10.1371/journal.pone.0024338

**Published:** 2011-08-31

**Authors:** Cornelia Hahn, Chong Wang, A. Wayne Orr, Brian G. Coon, Martin Alexander Schwartz

**Affiliations:** 1 Department of Microbiology, University of Virginia, Charlottesville, Virginia, United States of America; 2 Robert M. Berne Cardiovascular Research Center, University of Virginia, Charlottesville, Virginia, United States of America; 3 Department of Pathology, Louisiana State University Health Sciences Center, Shreveport, Louisiana, United States of America; 4 Department of Medicine, Yale University, New Haven, Connecticut, United States of America; 5 Mellon Prostate Cancer Research Center and Departments of Cell Biology and Biomedical Engineering, University of Virginia, Charlottesville, Virginia, United States of America; University of Pennsylvania School of Medicine, United States of America

## Abstract

Endothelial cells in straight, unbranched segments of arteries elongate and align in the direction of flow, a feature which is highly correlated with reduced atherosclerosis in these regions. The mitogen-activated protein kinase c-Jun N-terminal kinase (JNK) is activated by flow and is linked to inflammatory gene expression and apoptosis. We previously showed that JNK activation by flow is mediated by integrins and is observed in cells plated on fibronectin but not on collagen or basement membrane proteins. We now show thatJNK2 activation in response to laminar shear stress is biphasic, with an early peak and a later peak. Activated JNK localizes to focal adhesions at the ends of actin stress fibers, correlates with integrin activation and requires integrin binding to the extracellular matrix. Reducing JNK2 activation by siRNA inhibits alignment in response to shear stress. Cells on collagen, where JNK activity is low, align slowly. These data show that an inflammatory pathway facilitates adaptation to laminar flow, thereby revealing an unexpected connection between adaptation and inflammatory pathways.

## Introduction

Vascular endothelial cells (ECs) are exposed to high, unidirectional laminar shear stress in straight segments of arteries, whereas ECs at branch points and regions of high curvature experience disturbed flow, characterized by low shear stress, flow separation and flow reversal [1]. Disturbed flow is associated with inflammatory signaling and susceptibility to atherosclerosis, whereas high laminar shear stress is associated with a quiescent EC phenotype that is resistant to atherosclerosis [2]. Elongation of the cells and alignment of the actin stress fibers in the direction of flow is a hallmark of atheroprotected regions in vivo, whereas ECs in regions of high susceptibility to atherosclerosis are less elongated and poorly aligned. Cells in vitro exposed to high, laminar shear stress for long times also adapt to shear by adopting an elongated cell shape and aligning actin stress fibers in the direction of flow, and by downregulating inflammatory signaling pathways [3]. Indeed, evidence suggests a role for actin alignment in the downregulation of JNK [4].

Previous work has identified a mechanosensory complex consisting of VE-cadherin, VEGF receptor 2 and PECAM-1 at cell-cell junctions that is required for flow-dependent cell alignment and inflammatory activation [5]. Stimulation of this complex leads to phophoinositide-3-kinase activation and subsequent conversion of low affinity, unoccupied integrins to the high affinity, activated state. Newly activated integrins bind the subendothelial extracellular matrix, resulting in activation of small GTPases such as Rac, Rho and Cdc42 that mediate EC alignment and microtubule organizing center reorientation in response to laminar shear stress [6], [7], [8]. However, the effectors downstream of small GTPases that mediate this adaptation response are not fully understood.

Recent work in our lab showed that the mitogen activated protein kinase (MAPK), c-Jun N-terminal kinase (JNK) is activated by flow in a matrix-specific manner by onset of laminar shear stress [9]. In this system, ECs that were adhered to fibronectin activated JNK in response to flow whereas cells adhered to collagen or basement membrane protein did not, suggesting a link between matrix remodeling and inflammatory signaling. Interestingly, JNK has also been implicated in cytoskeletal reorganization in a number of systems, including cell migration and Drosophila dorsal closure during development [10], [11]. Consistent with these effects, active JNK can localize to focal adhesions and cytoskeletal structures [12], [13]. These data led us to consider whether activation of JNK could have a role in the alignment of endothelial cells under flow, as well as its role in inflammatory gene expression [14]. Here, we further characterize the upstream pathways by which JNK2 is activated by laminar shear stress and show that it is required for cell alignment.

## Results

### JNK2 activation by laminar shear is biphasic

**Figure 1:**
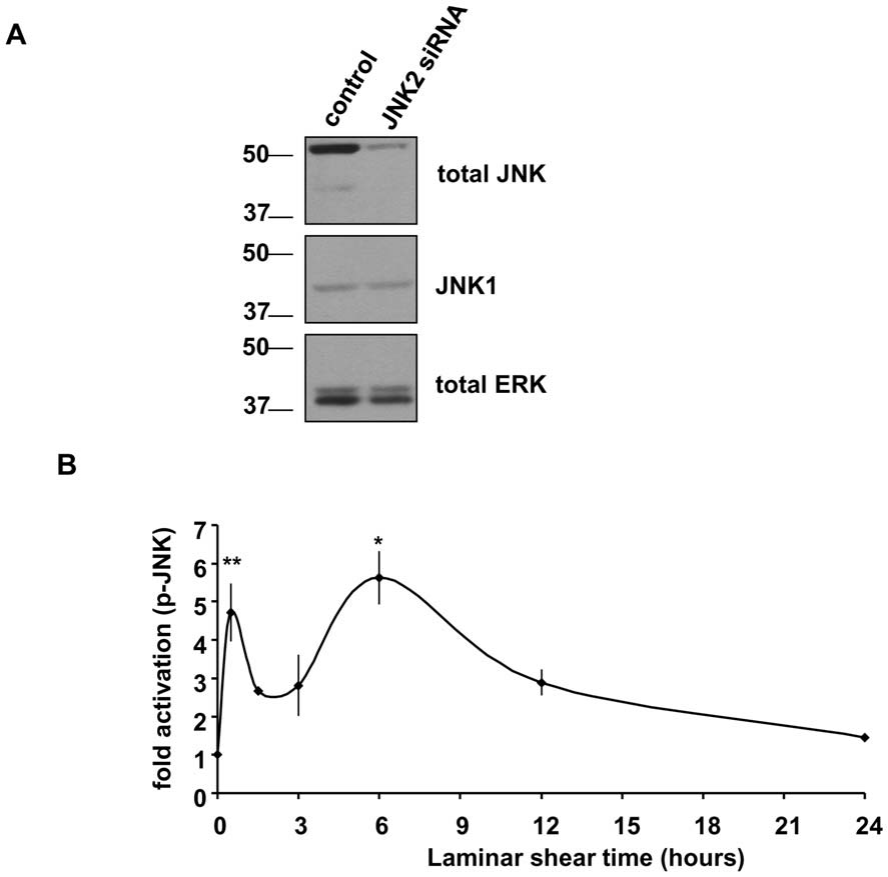
JNK2 activation on fibronectin is biphasic. (A) Representative Western blots showing BAECs transfected with either control or JNK2 siRNA. 48 hours after transfection, total cell lysates were immmunoblotted for total JNK and JNK1, using ERK as a loading control. (B) BAECs were exposed to laminar shear stress (12 dynes/cm^2^) for the indicated times. Total cell lysates were immunoblotted for activated phospho- Thr183/Tyr 185 JNK and total JNK. Values are means ± SEM after normalization to total levels (n = 3–4). *p<0.05**p<0.01.

Previous work showed that onset of fluid shear stress activated JNK [15], [16], [17], [18], however, these studies only examined short times. In bovine aortic endothelial cells (BAECs), phospho-specific and total JNK antibodies recognized a major band at 54 kD and a minor band at 46 kD, thought to correspond to JNK2 and JNK1, respectively [19]. Using siRNA targeted to JNK2, we confirmed that the major p54 band was indeed JNK2, and subsequent studies focused on JNK2 (Fig. 1A). We first characterized the activation of JNK2 by laminar shear over the entire time during which cells align in flow. BAECs plated on glass slides coated with FN were untreated or exposed to laminar shear stress (12 dynes/cm^2^) for up to 24 hours (Fig. 1B). Surprisingly, JNK2 activation was biphasic, with a first peak at around 0.5 h, followed by a second peak at 6 h that returned toward baseline by 24 hours.

### JNK activation in laminar shear is integrin dependent

**Figure 2:**
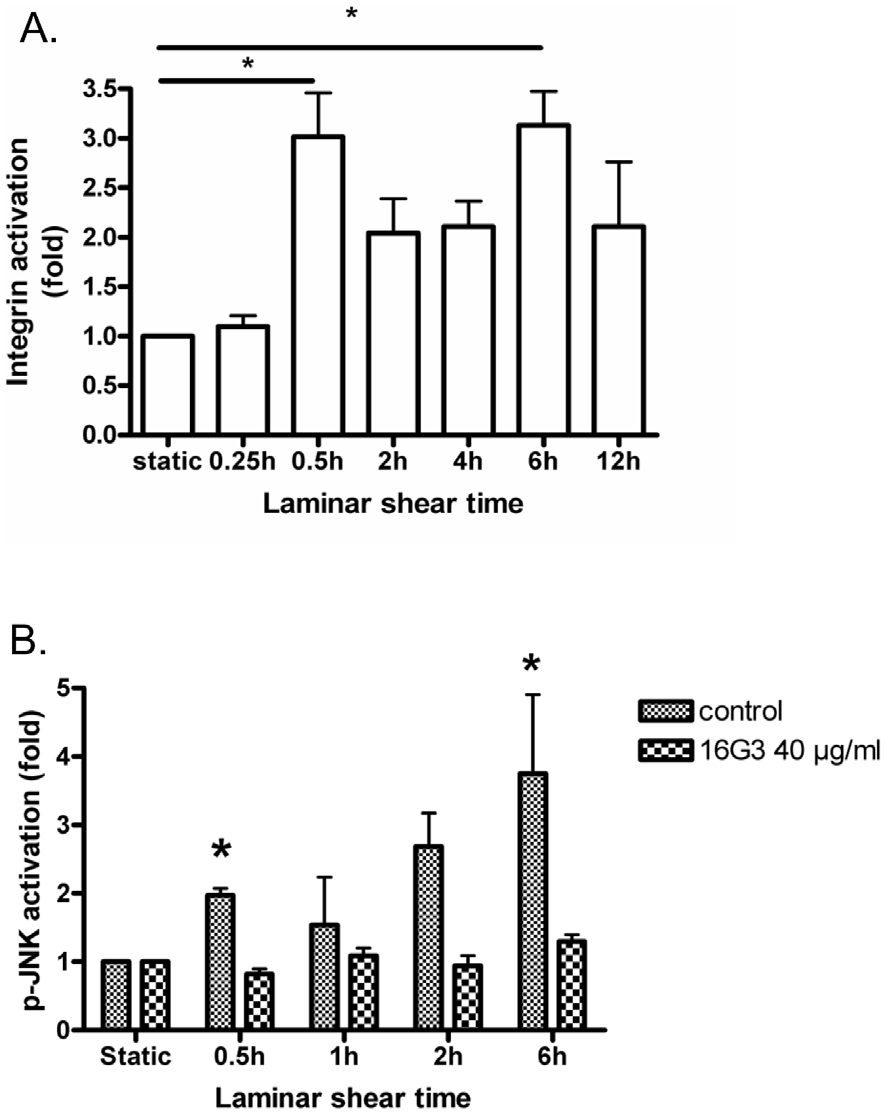
Integrin involvement in JNK2 activation by laminar shear stress. (A) Integrin activation. BAECs plated on FN were sheared for the indicated times. then GST-FNIII_9–11_ binding assayed as described in Methods. Total cell lysates were immunoblotted for GST and tubulin. Values were normalized to total cell material using tubulin as a standard. Values are relative to unstimulated cells. (B) Blocking FN. Cells were sheared in the presence of anti-FN antibody 16G3 at 40 µg/ml, then lysed and processes as in A. Values are means ± SEM (n = 3). *p<0.05.

A previous study showed that JNK activation was downstream of integrin activation and depended on the extracellular matrix (ECM) protein on which the cells were plated [9]. To test whether this pathway also mediates the late peak of JNK activity, we first examined integrin activation over a similar time frame. BAECs were exposed to flow as before and integrin α5β1 activation was assayed by measuring binding by a soluble fibronectin (FN) fragment [20]. We observed biphasic integrin activation with a time course that was similar to that of JNK activation (Fig. 2A). To test whether JNK2 activation requires new integrin binding, we used a blocking antibody 16G3 against FN; treatment with this antibody did not disrupt cell adhesion or cause loss of cells from the coverslips, as observed in previous studies [7], [20]. This antibody strongly suppressed both peaks of JNK activity (Fig. 2B). Together, these data show that JNK activation is biphasic due to biphasic integrin activation.

### JNK phosphorylation in focal adhesions with shear

**Figure 3:**
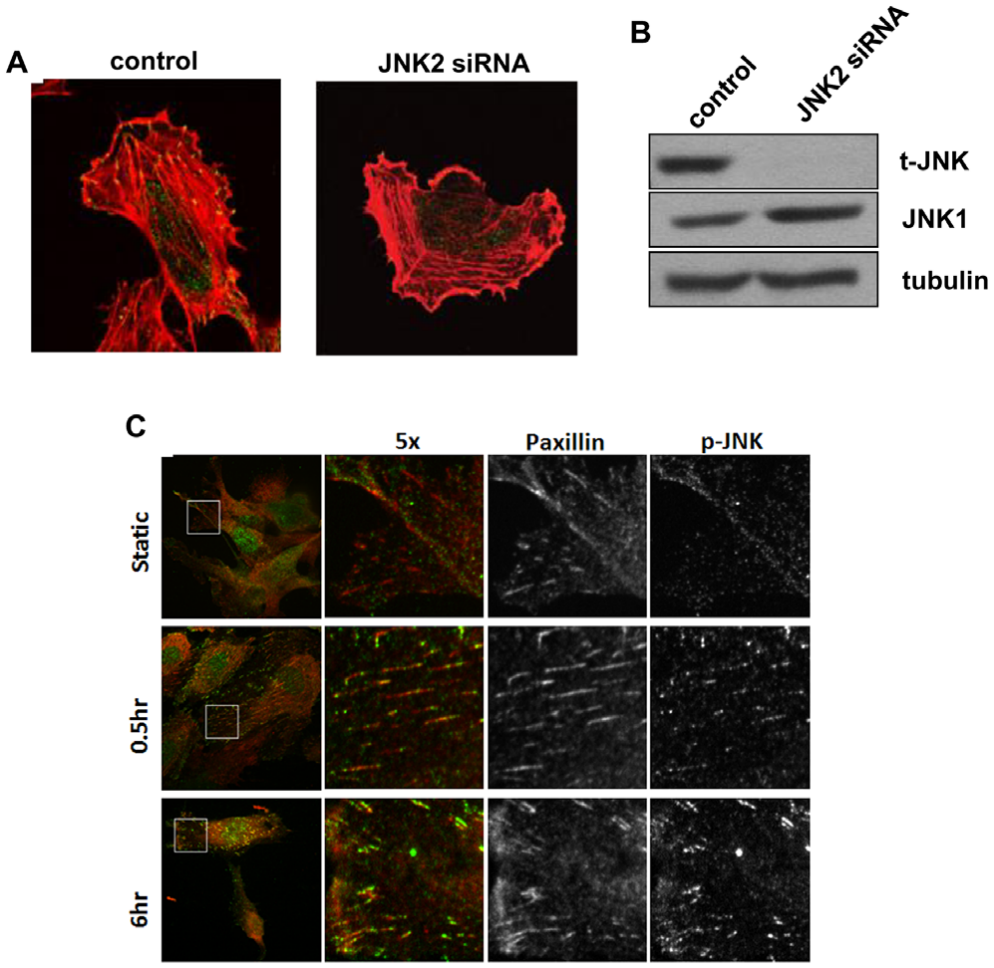
Activated JNK in focal adhesions. (A) Specific staining for activated JNK. HUVECs transfected with either control or JNK2 siRNA for 48 h were plated on FN-coated coverslips 2 hours, then fixed and stained for phospho-JNK (green) and actin (red). (B) Western blot of JNK2 knockdown. (C) HUVECs were left untreated or exposed to laminar shear stress as indicated. Cells were stained for phospho-JNK (green) and paxillin (red). Results are representative of 3 experiments.

JNK has previously been reported to localize to focal adhesions and cytoskeletal structures [13]. To investigate whether this was also the case in endothelial cells under flow, we used human umbilical vein endothelial cells (HUVECs). These cells also show biphasic activation of JNK in response to onset of laminar shear and are better suited for both antibody staining and RNAi. ([21] and data not shown) HUVECs were transfected with either control or JNK2 siRNA, then plated on FN coated coverslips and stained for phospho-JNK and actin. Cells transfected with control siRNA showed phospho-JNK staining in focal adhesion-like structures at peripheral ends of actin stress fibers, which was absent in cells transfected with JNK2 siRNA (Fig. 3A). Western blots showed almost complete loss of JNK2 after transfection, while JNK1 was unaffected (Fig. 3B). When cells exposed to laminar shear stress were examined, flow was observed to stimulate an increase in phospho-JNK focal adhesion staining (Fig. 3C). At all time points, cells still showed nuclear staining for phospho-JNK. BAECs also showed flow-stimulated phospho-JNK localization to focal adhesions (not shown).

### JNK2 is required for alignment in response to shear

**Figure 4:**
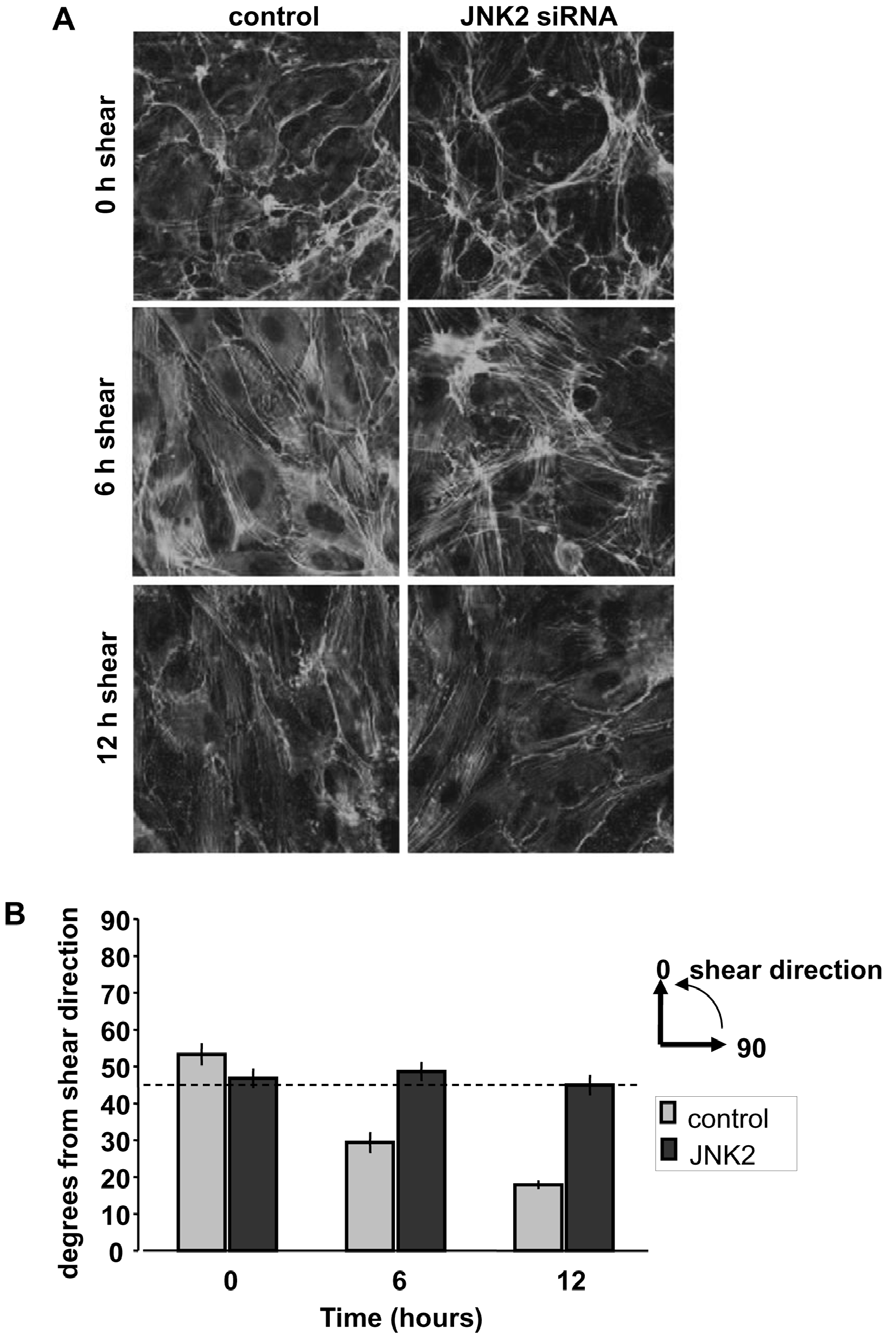
JNK2 is required for alignment in flow. (A) HUVECs on FN-coated glass slides were transfected with control or JNK2 siRNA. At 48 hours, cells were left untreated or exposed to flow as indicated. They were then fixed and stained for F-actin. (B) Alignment was quantified by measuring stress fiber angles from the direction of shear (taken to be 0 degrees). Three stress fibers were measured per cell, with 50–70 cells measured per condition (light gray = control siRNA, dark gray = JNK2 siRNA). Values are means ± SEM.

Given the involvement of JNK in focal adhesion and cytoskeletal remodeling [10], we next considered whether JNK might participate in EC alignment in the direction of flow [3]. HUVECs were transfected with either control or JNK2 siRNA, and exposed to flow (Fig. 4A,B). These results showed complete blockade of alignment following JNK2 depletion. Rescue using HA-JNK2 co-transfected with JNK2 siRNA was attempted, however, co-expression of even low levels of HA-JNK2 adversely affected cell survival, making it difficult to establish confluent cell monolayers (data not shown). Thus, to exclude off-target effects, alternate two JNK2 siRNA sequences, termed JNK2-2 and JNK2-3, were used. These JNK2 siRNA sequences also decreased JNK2 protein levels in HUVECs and inhibited endothelial cell alignment in response to shear stress (Fig. S1). Efficient inhibition by three different siRNA sequences provides strong evidence that JNK2 is specifically required for flow-mediated EC alignment.

### Matrix-specific JNK activation and cell alignment

**Figure 5:**
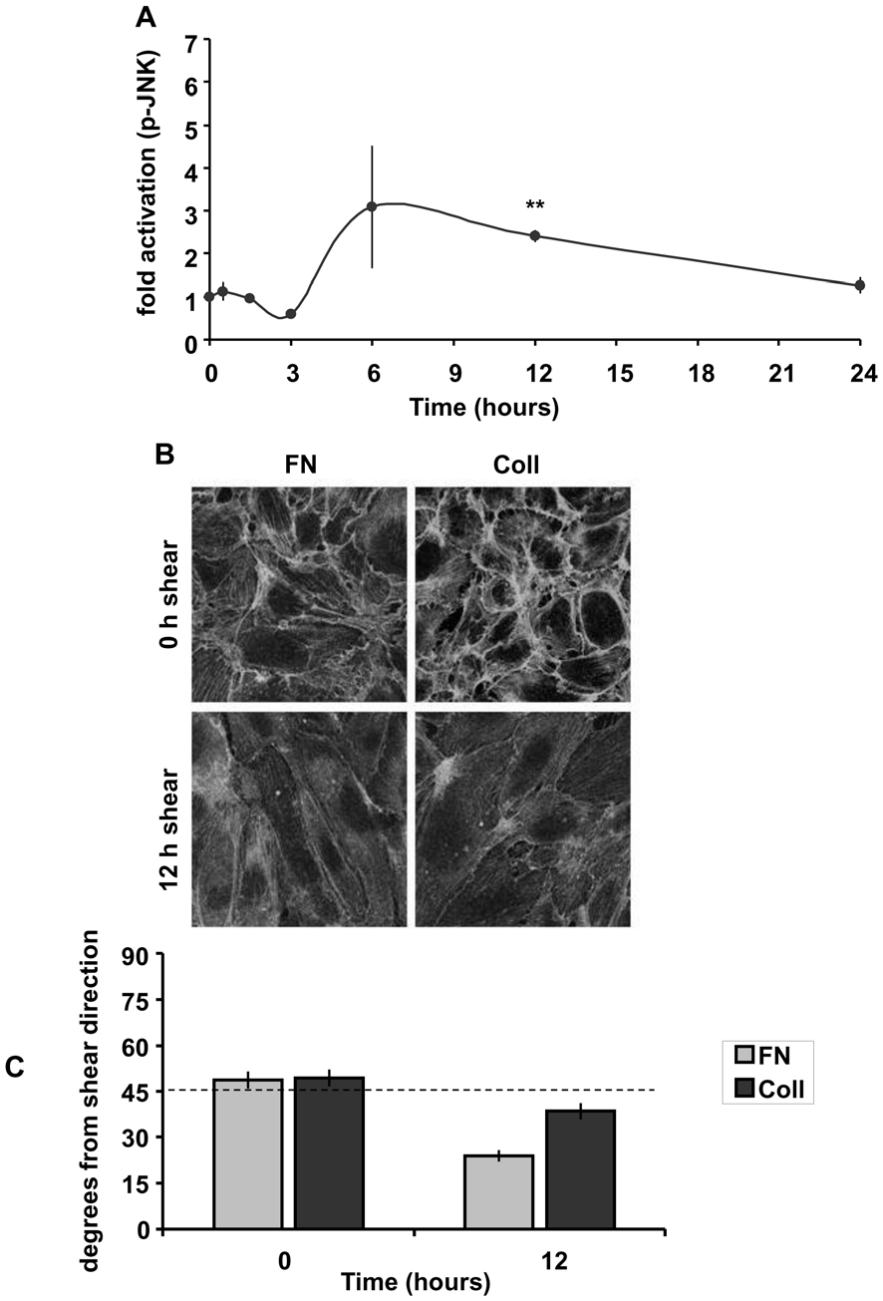
Collagen inhibits JNK activation and cell alignment. (A) BAECs plated on collagen overnight were untreated or exposed to laminar shear stress (12 dynes/cm^2^) as indicated. JNK activation was assayed as in Fig. 1. Values are means ± SEM (n = 3–4). **p<0.01 (B) HUVECs plated on either fibronectin-coated or collagen-coated glass slides were exposed to shear stress as indicated, then fixed and stained for F-actin. Images are representative of 3 experiments. (C) Alignment was quantified as in Fig. 4. Values are means ± SEM.

Our previous studies showed that JNK activation by flow is suppressed by adhesion of cells to collagen ^25^. This result led us to ask whether alignment was similarly affected. We first carried out a longer time course of JNK2 activation by shear on collagen. When BAECs on collagen were exposed to laminar shear stress for up to 24 hours, the initial peak of JNK activity was completely lacking (compare to cells on FN, Fig. 1B) as expected [9], while the second peak was reduced (Fig. 5A). When cell alignment was examined by fixing cells and staining for F-actin, cells on FN aligned faster than those on collagen (Fig. 5B,C). Thus, higher JNK activity correlates with faster alignment.

### Endothelial cell alignment is independent of the JNK-paxillin pathway

JNK phosphorylates paxillin at serine 178, which is reported to mediate effects on cell migration [10], [22]. We therefore tested the role of this potential JNK downstream pathway in EC alignment. Paxillin was knocked down in BAECs, then reconstituted with wide-type or nonphosphorylatable S178A paxillin. However, neither paxillin knockdown nor re-expression of mutant paxillin noticeably affected alignment under flow (Fig. S2). We conclude that JNK affects cell alignment independently of paxillin.

## Discussion

In this study, we report that JNK2 is activated by laminar shear stress in a biphasic manner, with peaks at approximately 0.5 and 6 hours, after which activity returns to baseline. Both peaks of JNK activity are downstream of integrin activation, which also has a two-peak activation profile. JNK activated through this pathway was concentrated in focal adhesions at the end of actin stress fibers and knockdown of JNK2 prevented alignment of HUVECs in response to flow. Cells on collagen also aligned more slowly than cells on FN consistent with lower JNK activation. JNK-mediated cell alignment is paxillin-independent.

While this manuscript was in preparation, a paper was published that reported that two JNK inhibitors blocked EC alignment under flow [23]. However, these inhibitors had distinct effects on basal cytoskeletal organization, raising doubts about the specificity of the observed effects. Our experiments using three different siRNA sequences to deplete JNK2 protein to demonstrate a specific effect on re-alignment under flow without obvious effects on basal cytoskeletal organization.

JNK activation by shear stress was previously linked to inflammatory gene expression [24]. In this context, activation of both JNK and downstream inflammatory genes by laminar shear were transient, whereas disturbed shear stimulates sustained activation, consistent with high activity in athero-susceptible regions of arteries in vivo [9]. These results support the idea that the initial response of ECs to onset of laminar shear is inflammatory, whereas at later times cells adapt and downregulate inflammatory pathways [25]. By contrast, in disturbed flow, the constant changes in shear stress magnitude and direction prevent adaptation, leading to sustained inflammatory signaling.

Our current results are consistent with this view but reveal an unexpected connection between the inflammatory and adaptive pathways. JNK2 activation by onset of laminar shear activates inflammatory signaling but also promotes the adaptive morphological response that downregulates inflammation. Only under disturbed flow where cells cannot adapt does JNK lead to sustained inflammatory signaling. These considerations also suggest a different perspective on matrix remodeling. Deposition of FN is induced transiently by onset of laminar shear and in a sustained manner by disturbed shear [26]. FN promotes a number of critical inflammatory events in addition to JNK, including activation of NF-κB and induction of leukocyte adhesion receptors. However, our current data show that FN also accelerates EC alignment in flow. These distinct effects can be reconciled if the inflammatory and alignment pathways have both evolved to facilitate vascular remodeling in response to changing flow conditions. Inflammation is a critical component of nearly all tissue remodeling processes [27], [28], [29]. Indeed, increased leukocyte recruitment contributes to vessel remodeling in several circumstances [27], [29]. Consistent with this model, inhibiting FN matrix assembly in mice blocked flow-dependent artery remodeling [30]. We speculate that under disturbed flow, these inflammatory pathways are activated but cannot promote remodeling to restore healthy laminar shear. Future work will be required to test this concept of maladaptive signaling under different flow patterns.

## Materials and Methods

### Cell culture, parallel plate flow chamber, and transfection

BAECs were groown in DMEM (Invitrogen), supplemented with 10% fetal bovine serum (FBS; Atlanta Biologicals), 10 U/ml penicillin and 10 µg/mL streptomycin (Invitrogen). Human umbilical vein endothelial cells (HUVECs) were maintained in DMEM:F12 media containing 10% FBS, 1% bovine brain extract, 60 µg/mL heparin (Sigma), 10 U/ml penicillin, and 10 µg/ml streptomycin. For shear stress experiments, cells were plated on glass slides coated with 20 µg/mL FN and allowed to form a confluent monolayer overnight. BAECs were starved for 4 hours in DMEM containing 1% FBS before being loaded onto a parallel plate flow chamber. HUVECs were starved in DMEM:F12 containing 2% FBS before being loaded onto a parallel plate flow chamber, and 12 dynes/cm^2^ of shear stress was applied for the indicated times.

Transient transfection of cells with control luciferase siRNA (5′-CGUACGCGGAAUACUUCGATT-3′), JNK2 SMARTpool siRNA (Thermo Scientific) and alternate JNK2 siRNA: JNK2-2 (5′-CAAGAAUGGUGUUGUAAAATT-3′), JNK2-3 (5′-GAUUUGAAGCCUAGCAACATT-3′) was accomplished using Lipofectamine 2000 (Invitrogen) according to manufacturer's protocol. Shear stress was applied 48 h after transfection. Transient transfection of cells with bovine paxillin knock-down SiRNA (5′-GGAUUACUUUGACAUGUUUUU-3′) was done using Lipofectamine RNAiMAX according to manufacturer's protocol, double knock downs were done in two consecutive days to ensure high knock-down efficiency. Paxillin plasmids for reconstitution were generously provided by Dr. Ken Jacobson (UNC Chapel Hill). They were used to transect BAECs using Lipofectamine 2000 one day after the second paxillin knock down. Shear stress was applied 24 h after the reconstitution transfection.

### Integrin activation and blocking assays

Activation state of integrin α5β1 was measured by a glutathione *S*- transferase (GST) fusion protein consisting of FN type III repeats 9–11 [31] as described [20]. Briefly, sheared or control cells on slides were washed with PBS, then incubated with 20 µg/ml GST- FNIII_9–11_ in PBS at 37° for 30 min. Cells were washed with PBS, lysed in SDS sample buffer, and bound GST-FNIII_9–11_ was assayed by western blotting for GST.

Blocking antibody 16G3, which blocks both α5β1 and α_V_β_3_ binding sites for FN, was used to inhibit the effects of integrin activation induced by shear stress. The cells were incubated with 20 or 40 µg/ml of 16G3 at 37° for 30 minutes before the onset of shear, and medium used for shear experiment contains the same concentration of 16G3 to ensure continuous blocking during the entire shear experiment.

### Immunoblotting

Cells were lysed in Laemmli buffer (0.1 M Tris pH 6.8, 10% β-mercaptoethanol, 20% glycerol, 4% SDS). Samples were run on 10% SDS-PAGE gels, transferred to PVDF membranes (BioRad) and blocked with 5% milk in TBS containing 0.01% Tween-20. Blots were incubated with 1∶1000 primary antibody total-ERK, phospho-JNK, total-JNK, (Cell Signaling Technology) or JNK1 (Affinity BioReagents) or 1∶10000 tubulin antibody overnight at 4°C. Blots were washed, incubated with 1∶5000 secondary goat anti-rabbit HRP antibody (Jackson Laboratories) or 1∶2500 goat anti-mouse HRP antibody (Jackson Laboratories) for 1 hour and developed using ECL reagents (Pierce) and film (Kodak).

### Immunocytochemistry

For the JNK/actin co-localization, cells were fixed in PBS containing 2% formaldehyde for 10 min., permeabilized with 0.5% Triton X-100 for 10 min., and blocked with PBS containing 10% goat serum (Atlanta Biologicals) for 1 hour at room temperature. Cells were incubated with rabbit phospho-JNK (BioSource) primary antibody at 5 µg/mL in blocking buffer for 3 hours at room temperature, followed by 1∶500 Alexa 488-conjugated phalloidin and 1∶1000 Alexa 568-conjugated goat anti-rabbit IgG (Molecular Probes) for 1 hour at room temperature. For phospho-JNK/paxillin co-localization, cells stained for phospho-JNK were then incubated with mouse monoclonal anti-paxillin antibody (BD Transduction) at a 1∶1000 dilution at 4 overnight, followed by washing and incubation with Alexa-fluor 568 labeled donkey anti-mouse secondary for 1 h at room temperature. For alignment experiments, cells were stained with 1∶500 Alexa 488-conjugated phalloidin for 1 hour at room temperature. Slides were mounted with Fluoromount G and images were captured using a Zeiss LSM510 scanning confocal microscope with a 63× oil immersion lens.

### Quantification and statistical analysis

Images were analyzed with Image J to calculate stress fiber angles. Immunoblots were quantified using Image J. Statistics were anlyzed using the two-tailed Student's t-test in Microsoft Excel. Statistical significance was taken as p<0.05.

## Supporting Information

Figure S1 **Additional JNK2 siRNA sequences.** A. HUVEcs were transfected with two different the siRNA oligos as in Figure 4 and Materials and Methods. Levels of JNK1 and 2 were assayed by Western blotting using tubulin as a loading control. B. Images of cells stained for F-actin at different times after application of shear. Results are representative of 3 independent experiments. C. Quantification of alignment. The orientation of the actin stress fibers was quantified as in Fig. 4, values are means ± SEM.
(TIF)

Figure S2 **Paxillin in EC alignment under flow.** A. Cells were transfected with a control siRNA oligo or siRNA directed against paxillin. Expression was assayed by Western blotting as described in Materials and Methods. B and C. Cells in which paxillin was depleted with siRNA (Fig. C) or depleted and then rescued with GFP fusions of WT or mutant S178A paxillin(B) were subject to flow for the indicated times. They were then fixed and stained for F-actin, and imaged for both GFP and F-actin. Images of representative of 3 independent experiments.
(TIF)

## References

[1] Malek AM, Alper SL, Izumo S (1999). Hemodynamic shear stress and its role in atherosclerosis.. Jama.

[2] Traub O, Berk BC (1998). Laminar shear stress: mechanisms by which endothelial cells transduce an atheroprotective force.. Arterioscler Thromb Vasc Biol.

[3] Girard PR, Nerem RM (1993). Endothelial cell signaling and cytoskeletal changes in response to shear stress.. Front Med Biol Eng.

[4] Boon RA, Leyen TA, Fontijn RD, Fledderus JO, Baggen JM (2010). KLF2-induced actin shear fibers control both alignment to flow and JNK signaling in vascular endothelium.. Blood.

[5] Tzima E, Irani-Tehrani M, Kiosses WB, Dejana E, Schultz DA (2005). A mechanosensory complex that mediates the endothelial cell response to fluid shear stress.. Nature.

[6] Tzima E, del Pozo MA, Shattil SJ, Chien S, Schwartz MA (2001). Activation of integrins in endothelial cells by fluid shear stress mediates Rho-dependent cytoskeletal alignment.. Embo J.

[7] Tzima E, Del Pozo MA, Kiosses WB, Mohamed SA, Li S (2002). Activation of Rac1 by shear stress in endothelial cells mediates both cytoskeletal reorganization and effects on gene expression.. Embo J.

[8] Tzima E, Kiosses WB, del Pozo MA, Schwartz MA (2003). Localized cdc42 activation, detected using a novel assay, mediates microtubule organizing center positioning in endothelial cells in response to fluid shear stress.. J Biol Chem.

[9] Hahn C, Orr AW, Sanders JM, Jhaveri KA, Schwartz MA (2009). The subendothelial extracellular matrix modulates JNK activation by flow.. Circ Res.

[10] Huang C, Rajfur Z, Borchers C, Schaller MD, Jacobson K (2003). JNK phosphorylates paxillin and regulates cell migration.. Nature.

[11] Xia Y, Karin M (2004). The control of cell motility and epithelial morphogenesis by Jun kinases.. Trends Cell Biol.

[12] Hamel M, Kanyi D, Cipolle MD, Lowe-Krentz L (2006). Active stress kinases in proliferating endothelial cells associated with cytoskeletal structures.. Endothelium.

[13] Almeida EA, Ilic D, Han Q, Hauck CR, Jin F (2000). Matrix survival signaling: from fibronectin via focal adhesion kinase to c-Jun NH(2)-terminal kinase.. J Cell Biol.

[14] Turjanski AG, Vaque JP, Gutkind JS (2007). MAP kinases and the control of nuclear events.. Oncogene.

[15] Go YM, Park H, Maland MC, Darley-Usmar VM, Stoyanov B (1998). Phosphatidylinositol 3-kinase gamma mediates shear stress-dependent activation of JNK in endothelial cells.. Am J Physiol.

[16] Jalali S, Li YS, Sotoudeh M, Yuan S, Li S (1998). Shear stress activates p60src-Ras-MAPK signaling pathways in vascular endothelial cells.. Arterioscler Thromb Vasc Biol.

[17] Li YS, Shyy JY, Li S, Lee J, Su B (1996). The Ras-JNK pathway is involved in shear-induced gene expression.. Mol Cell Biol.

[18] Li S, Chen BP, Azuma N, Hu YL, Wu SZ (1999). Distinct roles for the small GTPases Cdc42 and Rho in endothelial responses to shear stress.. J Clin Invest.

[19] Bogoyevitch MA (2006). The isoform-specific functions of the c-Jun N-terminal Kinases (JNKs): differences revealed by gene targeting.. Bioessays.

[20] Orr AW, Ginsberg MH, Shattil SJ, Deckmyn H, Schwartz MA (2006). Matrix-specific suppression of integrin activation in shear stress signaling.. Mol Biol Cell.

[21] Hu Y, Hochleitner BW, Wick G, Xu Q (1998). Decline of shear stress-induced activation of extracellular signal-regulated kinases, but not stress-activated protein kinases, in in vitro propagated endothelial cells.. Exp Gerontol.

[22] Kimura K, Teranishi S, Yamauchi J, Nishida T (2008). Role of JNK-dependent serine phosphorylation of paxillin in migration of corneal epithelial cells during wound closure.. Invest Ophthalmol Vis Sci.

[23] Mengistu M, Brotzman H, Ghadiali S, Lowe-Krentz L (2011). Fluid shear stress-induced JNK activity leads to actin remodeling for cell alignment.. J Cell Physiol.

[24] Sumara G, Belwal M, Ricci R (2005). “Jnking” atherosclerosis.. Cell Mol Life Sci.

[25] Hahn C, Schwartz MA (2008). The role of cellular adaptation to mechanical forces in atherosclerosis.. Arterioscler Thromb Vasc Biol.

[26] Feaver RE, Gelfand BD, Wang C, Schwartz MA, Blackman BR (2010). Atheroprone Hemodynamics Regulate Fibronectin Deposition to Create Positive Feedback That Sustains Endothelial Inflammation.. Circ Res.

[27] Jiang B, Liao R (2010). The paradoxical role of inflammation in cardiac repair and regeneration.. J Cardiovasc Transl Res.

[28] Heutinck KM, ten Berge IJ, Hack CE, Hamann J, Rowshani AT (2010). Serine proteases of the human immune system in health and disease.. Mol Immunol.

[29] Silvestre JS, Mallat Z, Tedgui A, Levy BI (2008). Post-ischaemic neovascularization and inflammation.. Cardiovasc Res.

[30] Chiang HY, Korshunov VA, Serour A, Shi F, Sottile J (2009). Fibronectin is an important regulator of flow-induced vascular remodeling.. Arterioscler Thromb Vasc Biol.

[31] Ramos JW, Whittaker CA, DeSimone DW (1996). Integrin-dependent adhesive activity is spatially controlled by inductive signals at gastrulation.. Development.

