# Supplementary figures and images for: JNK2 Promotes Endothelial Cell Alignment under Flow
Source: PLoS One. 2011 Aug 31;6(8):e24338. doi: 10.1371/journal.pone.0024338 (PMC3164210; doi:10.1371/journal.pone.0024338)

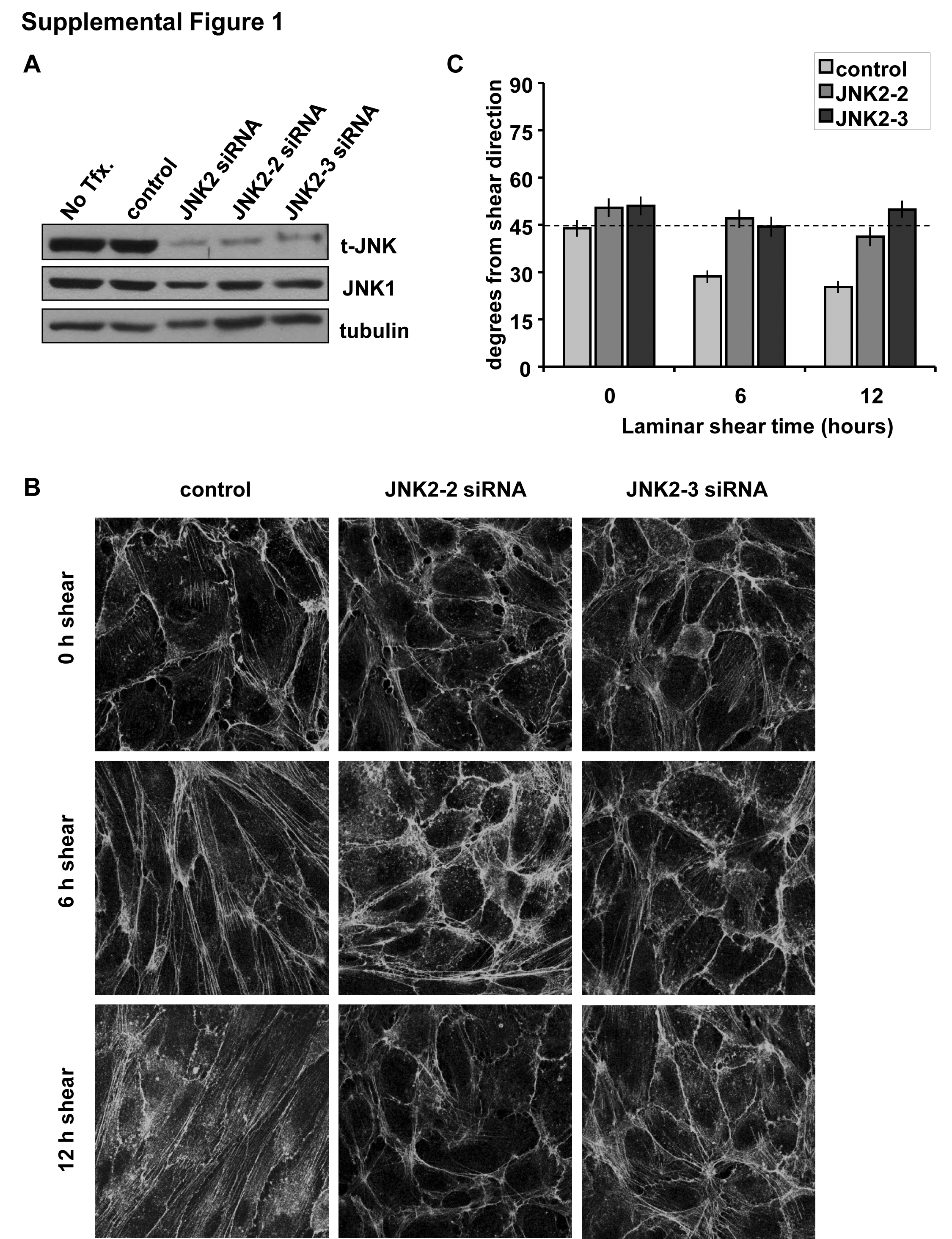

Supplement: Figure S1 — Additional JNK2 siRNA sequences. A. HUVEcs were transfected with two different the siRNA oligos as in Figure 4 and Materials and Methods. Levels of JNK1 and 2 were assayed by Western blotting using tubulin as a loading control. B. Images of cells stained for F-actin at different times after application of shear. Results are representative of 3 independent experiments. C. Quantification of alignment. The orientation of the actin stress fibers was quantified as in Fig. 4, values are means ± SEM. (TIF) [file pone.0024338.s001.tif]

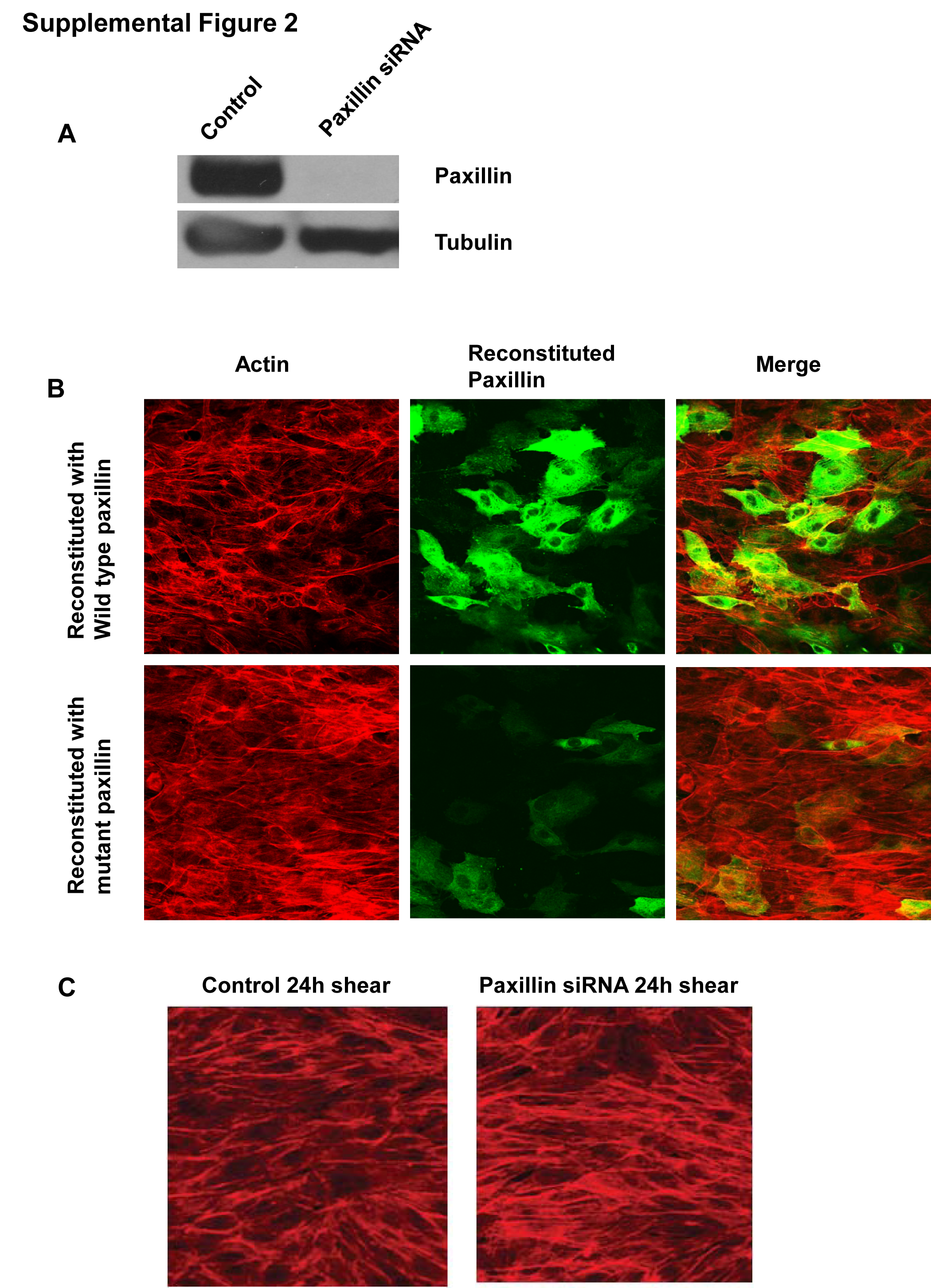

Supplement: Figure S2 — Paxillin in EC alignment under flow. A. Cells were transfected with a control siRNA oligo or siRNA directed against paxillin. Expression was assayed by Western blotting as described in Materials and Methods. B and C. Cells in which paxillin was depleted with siRNA (Fig. C) or depleted and then rescued with GFP fusions of WT or mutant S178A paxillin(B) were subject to flow for the indicated times. They were then fixed and stained for F-actin, and imaged for both GFP and F-actin. Images of representative of 3 independent experiments. (TIF) [file pone.0024338.s002.tif]
